# Serum Cystatin, Chemokine, and Gastrin-Releasing Peptide Precursors and Their Clinical Value in Patients with Chronic Renal Failure

**DOI:** 10.1155/2022/1775190

**Published:** 2022-01-21

**Authors:** Xiaoli Liu, Xin Liu, Jing Cai, Zhijie Xun, Qian Song, Ruixia Wang, Guiying Li, Zhongxin Xu

**Affiliations:** ^1^Department of Nephrology, Affiliated Hospital of Hebei Engineering University, Handan, Hebei, China; ^2^Affiliated Hospital of Hebei Engineering University, Handan, Hebei, China; ^3^Handan Central Hospital, Handan, Hebei, China; ^4^Handan First Hospital, Handan, Hebei, China; ^5^Handan Second Hospital, Handan, Hebei, China; ^6^Medical College, Hebei Engineering University, Handan, Hebei, China

## Abstract

**Objective:**

To investigate the serum cystatin (CysC), Chemerin, and gastrin-releasing peptide precursor (ProGRP) levels in patients with chronic renal failure (CRF).

**Methods:**

CRF patients admitted to our hospital from February 2019 to July 2019 were selected as the observation group, and 50 healthy patients were selected as the control group. The serum levels of CysC, Chemerin, ProGRP, and Scr of all subjects were detected. Patients with CRF were admitted for peritoneal dialysis (PD) treatment for 1 week, and continued treatment was performed. The survival rate of patients with CRF in nearly 1 year after continuous treatment was observed. Multivariate analysis of factors affecting survival time of CRF patients undergoing peritoneal dialysis was performed. The results were compared with those in the health group. The expression levels of CysC, Chemerin, ProGRP, and Scr in the observation group were all decreased, and the differences were statistically significant (*P* < 0.05). Pearson correlation analysis showed that Scr expression in CRF patients is positively correlated with CysC, Chemerin, and ProGRP (*P* < 0.001). The survival rate of 98 patients with CRF was 80.61% (79/98), and the mortality rate was 19.39% (19/98). Serum levels of CysC, Chemerin, ProGRP, and Scr in the death group are all higher than those in the survival group, and the differences are statistically significant (*P* < 0.05). CysC, Chemerin, ProGRP, and Scr are independent risk factors affecting survival time (*P* < 0.05). The AUC aspects of serum CysC, Chemerin, ProGRP, and Scr in predicting the survival rate of CRF patients in the treatment phase are 0.840, 0.775, 0.782, and 0.725, respectively.

**Conclusion:**

The serum levels of CysC, Chemerin, and ProGRP of CRF patients are abnormally elevated and are positively correlated with serum Scr of patients, which can be used as a reliable indicator of pathogenesis and prognosis assessment of CRF patients.

## 1. Introduction

Chronic renal failure (CRF) refers to the failure caused by slow renal decline because of various chronic renal parenchymal diseases [1, 2]. Due to the strong renal compensatory function, the early stage of CRF is not symptomatic. With the extension of time, renal function damage increases progressively, causing many complications, which may endanger the life of patients [3]. Early accurate diagnosis and effective treatment are very important to improve the prognosis of CRF patients. Previous diagnosis of CRF relied on serum creatinine (Scr), serum uric acid (SUA), blood urea nitrogen (BUN), and other renal function indexes, which often require kidney injury to account for more than half of the organ [4, 5] and is adverse to clinical diagnosis.

Renal function damage leads to abnormal glomerular filtration, which causes renal cell disorder, pH imbalance, and accumulation of many metabolites [6]. Cystatin C (CysC) is a small molecular weight protein that normally passes freely through the glomeruli [7]; renal function damage may lead to CysC accumulation. Chemerin is secreted by fat cells and can act on inflammatory response cells through the paracrine pathway [8]; a decrease in kidney function increases CysC levels. Gastrin-releasing peptide precursor (ProGRP) is produced in the gastrointestinal tract [9], which may induce the secretion of hormones in the digestive tract, increase nuclear heteromorphism, and possibly mediate renal injury. Therefore, understanding the serum CysC, Chemerin, and ProGRP changes of CRF patients may guide the early diagnosis of CRF. This study provides reference and guidance for early diagnosis of CRF by exploring the expression levels of serum CysC, Chemerin, and ProGRP.

## 2. Materials and Methods

### 2.1. General Information

CRF patients admitted to our hospital from February 2019 to July 2019 were selected as the observation group. Inclusion criteria include patients with complete clinical records and age > 18 years and (2) compliance with CRF diagnostic criteria and glomerular filtration rate (GFR) < 6 mL/min·1.73 m^2^. All the patients signed informed consent. Exclusion criteria include patients (1) complicated with heart, brain, and liver diseases, (2) complicated with infection and malignant tumor, and (3) with unstable blood sugar and blood pressure. A total of 98 patients met the inclusion criteria, including 66 males and 32 females, aged 55~76 years, with a mean of 59.69 ± 7.58 years old. The course of the disease was 2~12 years, with an average of 5.34 ± 1.56 years. Basic diseases include diabetic nephropathy in 18 cases, chronic glomerulonephritis in 69 cases, and nephrotic syndrome in 11 cases. 50 healthy people in our hospital were selected as the control group. There were no statistically significant differences in age and gender between the observation group and the control group (*P* > 0.05), as shown in Table 1.

### 2.2. Detection Methods

5 mL venous blood was collected on admission in the observation group, and 5 mL venous blood was collected on the day of physical examination in the control group. Blood samples were collected in a vacuum anticoagulant tube, gently shaken, and sent to the laboratory. Blood cells were removed by centrifuge, and serum Scr, CysC, and Chemerin levels were determined by ELISA. The serum ProGRP level was detected by a chemiluminescent microparticle immunoassay. The CysC detection kit was purchased from Chongqing Zhongyuan Biotechnology Co., LTD. The ProGRP detection kit was from Abbott. Scr and Chemerin detection kits were purchased from Seymour Fisher Technology (China) Co., LTD.

### 2.3. Treatment Methods

The CRF patients admitted to the hospital were routinely treated with anti-infection, hypoglycemic, and lipid-lowering drugs, electrolyte correction, and other conventional treatments. At the same time, peritoneal dialysis (PD) therapy (Baxter, USA) was used. The incision was made 1 cm from the midline of the navel, and the peritoneal dialysis tube was inserted into the vesicorectal fossa and then sutured. Intermittent peritoneal dialysis was performed for 1 week, followed by continuous treatment.

### 2.4. Grouping Methods

The survival rate of CRF patients after continuous treatment for nearly 1 year was observed, and the death group and the survival group were divided according to their survival rate.

### 2.5. Statistical Methods

SPSS 19.0 software was used for data processing, and the rate of enumeration data (%) was indicated. The chi-squared test was used. Measurement data were expressed as the mean ± standard deviation (±s), and a *t*-test was used. Pearson correlation analysis was used for correlation. Multivariate analysis of factors affecting survival time of CRF patients undergoing peritoneal dialysis was performed by logistic regression analysis. The survival rates of CRF patients in the treatment period are predicted by the ROC curve area under the curve (AUC) in CysC, Chemerin, ProGRP, and Scr indicators. *P* < 0.05 was considered statistically significant.

## 3. Results

### 3.1. Comparison of the Expression Levels of CysC, Chemerin, ProGRP, and Scr in the Observation Group and the Control Group

Compared with the health group, the expression levels of CysC, Chemerin, ProGRP, and Scr in the observation group were all decreased, and the differences were statistically significant (*P* < 0.05), as shown in Table 2.

### 3.2. Correlation Analysis of Serum Scr of CRF Patients with CysC, Chemerin, and ProGRP

Pearson correlation analysis showed that Scr expression in CRF patients is positively correlated with CysC, Chemerin, and ProGRP (*P* < 0.001), as shown in Table 3.

### 3.3. Serum Levels of CysC, Chemerin, ProGRP, and Scr in the Death and Survival Groups

The survival rate of 98 patients with CRF was 80.61% (79/98), and the mortality rate was 19.39% (19/98). Serum levels of CysC, Chemerin, ProGRP, and Scr in the death group are all higher than those in the survival group, and the differences are statistically significant (*P* < 0.05), as shown in Table 4. Multivariate analysis of factors affecting survival time of CRF patients undergoing peritoneal dialysis showed that CysC, Chemerin, ProGRP, and Scr were independent risk factors affecting survival time (*P* < 0.05), as shown in Table 5.

### 3.4. ROC Curve Analysis of Serum CysC, Chemerin, ProGRP, and Scr Indicators

**Figure 1:**
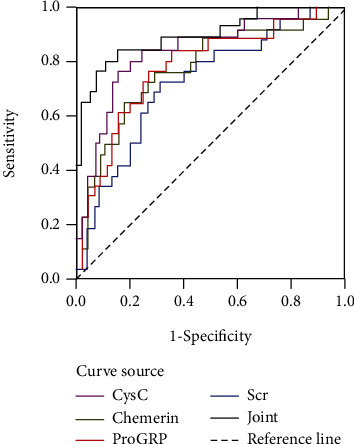
ROC curve analysis of serum CysC, Chemerin, ProGRP, and Scr indicators to predict the survival rate of CRF patients.

The AUC area of serum CysC, Chemerin, ProGRP, and Scr in predicting the treatment survival rate of CRF patients is 0.840, 0.775, 0.782, and 0725, respectively, of which the sensitivity and specificity of serum CysC, Chemerin, and ProGRP are higher than those of Scr indicators. See Figure 1 and Table 6.

## 4. Discussion

At present, the incidence of CRF in China is 38.4/1 million, and the prevalence rate has reached 853/1 million, which has become an important issue affecting the health of residents [10]. The development of CRF is irreversible, and the disease can only rely on renal replacement therapy to maintain life at a certain stage. Scr is a common indicator for clinical diagnosis of renal function. When renal function is impaired, the level of Scr will increase, but Scr is easily affected by diet, infection, drugs, and other diseases. In addition, when the kidney injury is not serious, the abnormal changes in Scr are not significant, which limited the early diagnosis of CRF [11, 12]. Therefore, it is necessary to develop more clinical indicators to evaluate CRF.

CysC is a low molecular weight basic nonglycosylated protein, which is produced at a constant rate in human nucleated cells. It does not bind to plasma proteins after production and is stable in the blood. The liver is the main organ for the clearance of CysC, which is metabolized near the convoluted tubule and filtered through the glomerulus. Therefore, the CysC level can reflect renal function [13]. Previous studies reported that changes in serum CysC occur when the glomerular basement membrane is slightly altered [14], suggesting that CysC may have certain diagnostic potential for early renal dysfunction. Chemerin is a chemokine, which can bind chemerin receptors on macrophages and stimulate the release of a variety of inflammatory mediators, such as tumor necrosis factor, interleukin, and chemokine [15–17]. In addition, chemerin could stimulate mesangial cells to secrete extracellular matrix proteins, which promotes mesangial proliferation and affects the barrier function of the glomerular capillary wall. Therefore, the glomerular filtration rate is independently related to the level of chemerin, and highly expressed chemerin can promote the occurrence and progression of CRF [18–20]. This suggests that the chemerin level is associated with renal function and could be used for early diagnosis of renal dysfunction. ProGRP is a precursor of gastrin-releasing peptide with a stable structure and long half-life. In humans, kidneys have the main metabolic pathways for ProGRP. Elevated serum ProGRP may be associated with renal excretion disorders and metabolic disorders. The results of this study show that serum levels of CRF patients in CysC, Chemerin, ProGRP, and Scr are significantly higher than those in healthy populations. Further analysis found that serum Scr of CRF patients is positively correlated with CysC, chemerin, and ProGRP, suggesting that levels of these serum proteins are correlated with the degree of kidney injury. After monitoring the survival rate of CRF patients in nearly 1 year of continuous treatment, it is found that serum levels of dead patients in CysC, chemerin, ProGRP, and Scr are significantly higher than those in survived patients, indicating that serum levels of CysC, Chemerin, ProGRP, and Scr in CRF patients on the day of hospital admission may affect the treatment response and survival rate of patients. After drawing the ROC curve to evaluate the efficiency of each indicator, it is found that the sensitivity and specificity of CysC, Chemerin, and ProGRP are better than those of Scr, likely because of the powerful renal function. A unilateral kidney is capable of maintaining Scr at the normal level, leading to its low sensitivity and specificity.

In summary, serum CysC, chemerin, and ProGRP of CRF patients are abnormally elevated and are positively correlated with serum Scr of patients. The sensitivity and specificity of serum CysC, chemerin, and ProGRP in predicting the survival rate of CRF patients in the treatment stage are better than those of Scr indicators. However, the cases analyzed in this study were only from our hospital, which belongs to a single-center study, and the results inevitably have some bias. Multicenter studies will be conducted in the future to further enhance the reliability of the research results.

## Figures and Tables

**Table 1:** Comparison of general data between the observation group and the control group.

Group	Case number	Age	Gender (male/female)
Control group	50	58.17 ± 8.91	33/17
Observation group	88	59.69 ± 7.58	56/32
*t*/*X*^2^		1.062	0.078
*P*		0.290	0.780

**Table 2:** Comparison of the expression levels of CysC, Chemerin, ProGRP, and Scr in the observation group and the control group.

Group	Number of cases	CysC (mg/L)	Chemerin (*μ*g/L)	ProGRP (pg/mL)	Scr (mu mol/L)
Control groups	50	0.51 ± 0.09	76.45 ± 5.65	27.43 ± 5.76	117.56 ± 16.72
Observation group	98	1.83 ± 0.45	107.61 ± 17.54	49.65 ± 10.43	179.14 ± 22.51
*t*		20.500	12.220	14.001	17.080
*P*		<0.001	<0.001	<0.001	<0.001

**Table 3:** Correlation analysis of serum Scr with CysC, Chemerin, and ProGRP in CRF patients.

Scr correlation	*r*_*s*_ value	*P* values
CysC	0.647	<0.001
ProGRP	0.736	<0.001
Chemerin	0.561	<0.001

**Table 4:** Comparison of serum levels of CysC, Chemerin, ProGRP, and Scr in the death group and the survival group.

Group	Number of cases	CysC (mg/L)	Chemerin (*μ*g/L)	ProGRP (pg/mL)	Scr (mu mol/L)
Death	19	2.29 ± 0.39	145.41 ± 18.75	61.75 ± 9.43	230.6 ± 18.51
Survival	79	1.72 ± 0.32	98.52 ± 14.92	46.74 ± 7.62	166.7 ± 16.63
*t*		6.674	11.680	7.351	14.710
*P*		<0.001	<0.001	<0.001	<0.001

**Table 5:** Multivariate analysis of factors influencing survival time of CRF patients undergoing peritoneal dialysis.

Index	*β*	SE	Wald	*P*	OR	95% CI
CysC (mg/L)	0.007	0.004	1.444	0.231	1.055	0.996~1.015
Chemerin (*μ*g/L)	0.991	0.211	20.232	<0.01	2.713	1.754~4.178
ProGRP (pg/mL)	0.456	0.088	37.307	<0.01	1.654	1.393~1.905
Scr (mu mol/L)	0.003	0.001	24.456	<0.01	1.032	1.002~1.006

Note: CysC, Chemerin, ProGRP, and Scr are continuous variables.

**Table 6:** ROC parameters of serum CysC, Chemerin, ProGRP, and Scr in CRF patients.

Indicators	AUC	Cutoff value	Sensitivity (%)	Specificity (%)	*P* values	95% CI
CysC	0.840	1.95 mg/L	83.30	88.50	*P* < 0.001	0.705-0.953
Chemerin	0.775	115.65 *μ*g/L	75.40	81.40	*P* < 0.001	0.614-0.872
ProGRP	0.782	53.45 pg/mL	80.50	87.60	*P* < 0.001	0.582-0.905
Scr	0.725	185.3 *μ* mol/L	70.70	77.40	*P* < 0.001	0.602-0.918

## Data Availability

The datasets used during the present study are available from the corresponding author upon reasonable request.
